# Computational screening and molecular dynamics of natural compounds targeting the SH2 domain of STAT3: a multitarget approach using network pharmacology

**DOI:** 10.1007/s11030-024-11075-5

**Published:** 2025-01-09

**Authors:** Sachindra Kumar, B. Harish Kumar, Raksha Nayak, Samyak Pandey, Nitesh Kumar, K. Sreedhara Ranganath Pai

**Affiliations:** 1 Department of Pharmacology, Manipal College of Pharmaceutical Sciences, Manipal Academy of Higher Education (MAHE), Manipal, 576104 India; 2 Department of Pharmacology and Toxicology, National Institute of Pharmaceutical Education and Research, Hajipur, Vaishali, Bihar 844102 India

**Keywords:** STAT3, Molecular docking, SH2 domain, Network pharmacology, Phytochemical, Cancer

## Abstract

SH2 (Src Homology 2) domains play a crucial role in phosphotyrosine-mediated signaling and have emerged as promising drug targets, particularly in cancer therapy. STAT3 (Signal Transducer and Activator of Transcription 3), which contains an SH2 domain, plays a pivotal role in cancer progression and immune evasion because it facilitates the dimerization of STAT3, which is essential for their activation and subsequent nuclear translocation. SH2 domain-mediated STAT3 inhibition disrupts this binding, reduces phosphorylation of STAT3, and impairs dimerization. This study employed an in silico approach to screen potential natural compounds that could target the SH2 domain of STAT3 and inhibit its function. The phytomolecules (182455) were retrieved from the ZINC 15 database and were docked using various modes like HTVS, SP, and XP. The phytomolecules exhibiting higher binding affinity were selected. MM-GBSA was performed to determine binding free energy, and the QikProp tool was utilized to assess the pharmacokinetic properties of potential hit compounds, narrowing down the list of candidates. Molecular dynamics simulations, thermal MM-GBSA, and WaterMap analysis were performed on compounds that exhibited favorable binding affinities and pharmacokinetic characteristics. Based on docking scores and binding interactions, ZINC255200449, ZINC299817570, ZINC31167114, and ZINC67910988 were identified as potential STAT3 inhibitors. ZINC67910988 demonstrated superior stability in molecular dynamics simulation and WaterMap analysis. Furthermore, DFT was performed to determine energetic and electronic properties, and HOMO and LUMO sites were predicted for electronic structure calculation. Additionally, network pharmacology was performed to map the compounds’ interactions within biological networks, highlighting their multitarget potential. Compound-target networks elucidate the relationships between compounds and multiple targets, along with their associated pathways and help to minimize off-target effects. The identified lead compound showed strong potential as a STAT3 inhibitor, warranting further validation through in vitro and in vivo studies.

**Graphical abstract:**

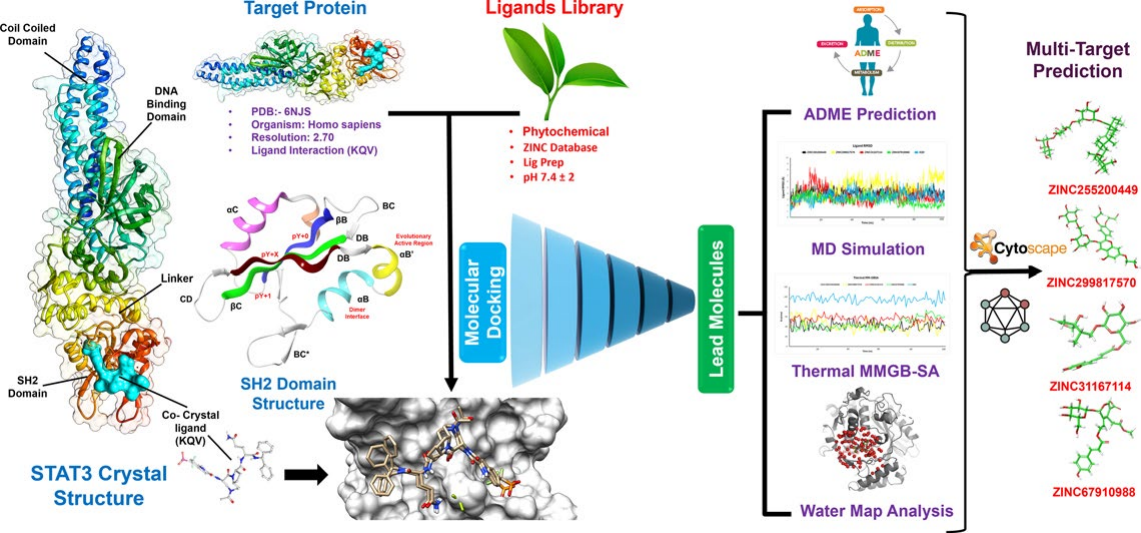

**Supplementary Information:**

The online version contains supplementary material available at 10.1007/s11030-024-11075-5.

## Introduction

STAT3 is a key transcription factor that regulates cell growth, survival, and differentiation [1]. Dysregulated activation of STAT3 has been directly linked to various human cancers, making it a significant target for cancer therapy [2]. Constitutive STAT3 activation is observed in many cancer types, including breast, prostate, lung, and hematological malignancies [3]. This STAT3 activation is often driven by sustained cytokine signaling, such as interleukin-6 (IL-6), and various growth factors, such as VEGF, EGF, and PDGF [4]. Once activated, STAT3 translocates to the nucleus, where it binds to DNA sequences, promoting gene expression in growth and survival [5]. This uncontrolled activation contributes to tumor formation, making STAT3 a crucial target for developing anticancer therapies [6].

Most instances involve the activation and dimerization of STAT3 protein through its SH2 (Src Homology 2) domain. This domain binds to a phosphorylated tyrosine residue (Y705) of another STAT3 molecule to form an active state dimer [7]. Disrupting this interaction has recently been the target for developing STAT3 inhibitors [8]. Arg 609, Glu 594, Lys 591, Ser 636 Ser 611, Val 637, Tyr 657, Gln 644, Thr 640, Glu 638, and Trp 623 are some amino acid residues which are showing direct or indirect binding involvement with the phosphoserine motif of STAT3 [9–13]. Mutations and disruptions in these amino acids can attenuate STAT3 signaling and activation.

**Fig. 1:**
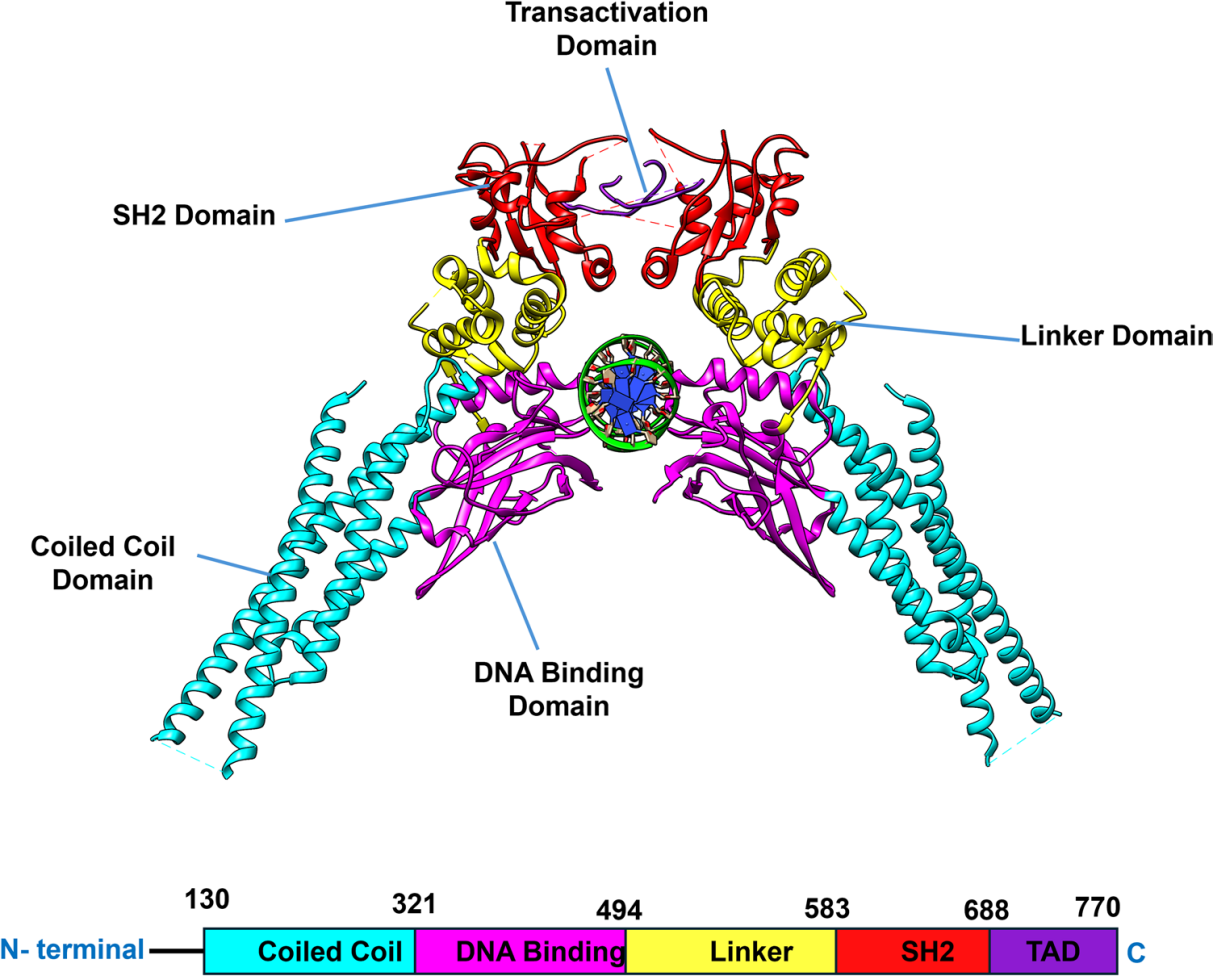
Structural organization of STAT3 [90, 91]

The SH2 domain structure is made up of two α-helices (αA and αB) sandwiched between a central anti-parallel β-sheet (containing the three β-strands typically labeled βB-βD), which is commonly known as the αβββα motif [14]. The pY binding pocket of the SH2 domain is divided into three sub-pockets referred to as the pY + X (hydrophobic side), pY + 0 (binds to pY705), and pY + 1 (binds to L706) pockets [15, 16]. The structural organization of STAT3 is shown in Fig. 1. The pY + 0 pocket interacts with the phosphotyrosine705 on STAT3 to stabilize dimerization and facilitate translocation of phosphorylated STAT3 to the nucleus. Following its translocation to the nucleus, STAT3 acts as a transcription factor and stimulates genes required for cell proliferation and survival [15, 17]. Stattic and SD36 are well-known small-molecule inhibitors designed to target the Src Homology 2 (SH2) domain of STAT3 [18, 19]. These compounds effectively disrupt STAT3 activation, dimerization, and nuclear translocation, inhibiting its oncogenic functions.

In silico screening techniques, especially molecular docking, have emerged as a potential strategy for hit identification in drug discovery [20–22]. The computational approach allows the virtual screening of huge compound libraries against target protein(s) to predict binding affinities and interaction modality [23, 24]. These methodologies have significantly streamlined research by enabling researchers to simulate biological interactions and drug properties based on molecular structures. Computational approaches enhance the efficiency of drug identification and reduce reliance on traditional experimental methods. Molecular docking is an effective method for screening natural products as potential inhibitors of the STAT3-SH2 domain [25]. Natural products have long been valuable sources of bioactive compounds, many of which possess distinct structures and mechanisms of action. With the help of computational methods, researchers can effectively identify potential inhibitors from extensive databases of natural compounds, thereby accelerating the drug discovery process [26–28].

Phytochemicals and natural compounds derived from plants, fungi, and other organisms have a myriad of pharmacological activities, many of which serve as anticancer agents [29, 30]. These compounds can often interact with selective molecular targets and modulate several cancer-related signaling pathways. Due to their inherent structural diversity and biological relevance, natural compounds are increasingly screened in molecular docking studies [31]. Historical success in drug discovery and therapeutic applications highlights that approximately 40% of FDA-approved drugs are derived from natural sources [32, 33]. Natural products can quickly evolve through biological processes, resulting in their being well suited for interacting with biological targets, often exhibiting higher specificity and potency than synthetic molecules [34, 35]. Its unique structural diversity, favorable pharmacological properties, historical efficacy as drugs, suitability for phenotypic screening, and potential for novel interactions with biological targets enhance insights in clinical translation. The investigation of natural substances as potential inhibitors of STAT3 is gaining momentum as they often exhibit better pharmacokinetic profiles and lower toxicity compared to synthetic drugs [36].

Moreover, network pharmacology provides an integrated perspective of the complex interplay between natural compounds and biological systems [37]. In contrast, by mapping interactions of compounds with targets and pathways, researchers can devise multitarget strategies that may increase therapeutic efficacy against the disease while reducing side effects [38]. The present study identifies new molecules showing good binding affinity and stability with the SH2 domain of STAT3 [39]. The identified hit might be a potential STAT3 inhibitor; however, more in vitro and in vivo testing is needed to validate this study.

## Materials and methods

The computational studies were conducted using the Maestro Schrödinger suite version 2024-2 (Schrödinger, LLC, New York) on a Linux-based workstation with 8 GB of RAM. For the study, programs including Protein Preparation Wizard, Ligprep, GLIDE, Desmond, Prime, and WaterMap were utilized.

### Protein selection and preparation

The Protein Data Bank (PDB) was explored for STAT3 crystal structures, and only two structures contained co-crystalized ligands, which were 6NJS and 6NUQ, making them suitable for docking. 6NJS was chosen as it had better resolution (6NJS 2.70 Å vs. 6NUQ 3.15 Å), lacked mutations in the SH2 domain, and had fewer gaps in its sequence. The protein was processed using the ‘Protein Preparation Wizard’ in the Schrodinger suite [40]. This wizard assists in identifying and rectifying any imperfections in the protein structure by incorporating hydrogen atoms, filling in missing side chains, and assigning bind orders. The downloaded protein was subjected to several processes, including import and refine, review and modification, and minimization processes. The missing residues and side chains were filled using the prime tool. The crucial binding pocket was left unaltered in the protein structure. The OPLS3e (Optimized potential for liquid simulation) force field was employed to minimize protein energy, resulting in a low-energy state protein.

### Database preparation

A total of 182,455 natural compounds were retrieved from the Zinc 15 compounds database (applied now availability criteria) [41, 42]. These compounds were obtained and processed to create suitable three-dimensional structures with optimized ionization states at a pH level of 7.4 ± 0.5 using the Ligprep tool from the Maestro suite. The resulting three-dimensional structures were utilized to examine the molecules’ chirality, and further optimization was carried out using the Optimized Potentials for Liquid Simulations force field.

### Ligand docking

The GLIDE tool, a grid-based ligand docking tool that helps to identify favorable interactions between the ligand and the protein, was utilized for all of the docking experiments [43]. The Receptor grid generation tool was used to create a grid in the place of the co-crystallized ligand, which represents the properties of the targeted protein and shape, coordinates of grid box *X*:13.22, *Y*:56.39, and *Z*:0.27 (length of ligands dock was 20 Å). To validate the receptor grid file, the inbound ligand was redocked into the created grid, and the root-mean-square deviation (RMSD) of posture generated before and after the docking was measured. This grid was used to generate precise scoring of the ligand poses. Initially, high-throughput screening (HTVS) was used for screening 182,455 prepared ligands, followed by Standard Precision (SP) docking of the top 55,872 molecules based on HTVS score. Finally, the Extra Precision (XP) docking model was executed for the top-scoring compounds (cut-off at − 6.5 kcal/mol) obtained through the SP mode, considered the most accurate method.

### Molecular mechanics generalized born surface area (MM-GBSA) analysis

The Prime MM-GBSA module determines protein–ligand complexes binding free energy (ΔG Binding) [44]. The OPLS3e force field and VSGB solvent model were used. This module combines molecular mechanics and solvation model calculations with a continuous system to calculate the binding free energy of the ligand–protein complex. In the equation provided below

$$ \Delta {\text{GBinding}} = \Delta {\text{GComplex}}{-}\left( {\Delta {\text{Greceptor}} + \Delta {\text{Gligand}}} \right) $$

ΔGBinding, ΔGreceptor, and ΔGligand denote the total binding energy of the complex, free receptor, and unbound ligand, respectively. More substantial binding potential is indicated by more negative kcal/mol values.

### Predicted pharmacokinetic property

The QikProp module was employed to predict the ADME properties of the chosen phytochemicals. Evaluation of the drug-likeness of the phytoconstituents was conducted using various descriptors and pharmacokinetic properties, including the predicted aqueous solubility (QPlogS), predicted apparent Caco-2 permeability (QPPCaco), predicted octanol/water partition coefficient (QPlogPo/w), and Lipinski’s rule of 5. Additionally, human oral absorption was considered in the evaluation. Using pkCMS webtool (https://biosig.lab.uq.edu.au/pkcsm/prediction), we predicted the toxicity profile of all compounds [45].

### Induced fit docking

Maestro was used to carry out induced fit docking (IFD) on the chosen ligands that were acquired by molecular docking. Unlike in physiological settings, the rigidity of the amino acid’s interaction with ligands is observed in docking experiments. Consequently, IFD is employed to allow for protein flexibility. The van der Waals scaling of the ligand and receptor was kept at 0.50 during IFD. A maximum of twenty poses of the ligand with protein were generated by the calculations, which were carried out using the normal precision technique. Each stance was examined separately, and the pose that showed the greatest number of interactions with important residues was chosen to be used in the molecular dynamic simulation.

### Molecular dynamics (MD) simulation

To understand the interactions between the chosen molecules and the STAT3 protein, molecular dynamics simulations were carried out using the Desmond module of the Schrodinger suite. Initially, the compound-6NJS protein complex was processed using the system builder module to make it suitable for simulation by solvating it in the predefined SPC solvent under orthorhombic boundary conditions (10 × 10 × 10 Å), OPLS4 force field. Subsequently, these solvated complexes were subjected to energy minimization until they reached a gradient threshold of 25 kcal/mol/A0. Finally, the solvated system was used in MD simulation at NPT (normal pressure and temperature) 1.01 bar pressure and 300 K temperature. The prepared system was then subjected to MD simulation for 500 ns. All the simulations were performed using the Desmond module. A simulated interaction diagram was generated to analyze the data.

To determine the binding energy of the ligands in various frames, MM-GBSA and MD simulation module were utilized. The MD simulation generated 1002 frames, and the binding energy was calculated for each tenth frame. The formula are used to compute the ligands' binding energies given below.

$$ {\text{MMGBSA dG Bind }}\left( {{\text{NS}}} \right) \, = {\text{ Complex-Receptor }}\left( {\text{from optimized complex}} \right) \, {-}{\text{ Ligand }}\left( {\text{from optimized complex}} \right) $$

### WaterMap analysis

The WaterMap tool [31] from Maestro was employed to investigate the function of water molecules in the protein's binding place. For this investigation, we examined water molecules within a 10-angstrom radius surrounding the location where the ligand was bound in the protein (6NJS). The crystallographic water molecules in the protein were eliminated prior to the simulation, which used the OPLS3e force field and lasted ten nanoseconds. Next, the possible hydration sites and stable binding affinities of each ligand that made the shortlist were examined.

### Density-functional theory (DFT)

The density-functional theory (DFT) method is used to calculate Single Point Energy (SPE), which displays the electronic properties and energies of compounds at the atomic level. All compounds’ electronic characteristics have been calculated using Schrodinger’s Jaguar module (Schrödinger Release 2024-2). Using the hybrid DFT with the default B3LYP (Berke's three-parameter exchange potential and Lee–Yang–Parr correlation function) basis set 6-31 G**, electronic characteristics found in the compounds were derived by geometry improvements. The energy is computed using the Adaptive Poisson–Boltzmann Finite (PBF) solvation under physiological conditions. Maestro version 14.0.134 is used to illustrate the DFT's calculations of the aqueous solvation energy, the lowest unoccupied molecular orbital (LUMO), and the highest occupied molecular orbital (HOMO) [46].

### Network pharmacology

After molecular docking and MD simulation, network pharmacology was performed to elucidate the multitarget nature of compounds by mapping interactions across biological networks. It integrates data from molecular interactions, signaling pathways, gene regulation, and disease associations. In this study, the potential genes related to compounds were retrieved from the Superpred target prediction (https://prediction.charite.de/subpages/target_prediction.php) web tool and 84 compound-related genes were obtained. 18,007 genes related to cancer were obtained from GeneCards (https://www.genecards.org/) by applying a filter 30 GeneCards Inferred Functionality Scores (GIFtS) specific criteria. The Venny 2.1.0 web tool, available at (https://bioinfogp.cnb.csic.es/tools/venny/) was utilized to determine the overlap between cancer targets and compound targets. Targets that overlapped were added to STRING (https://string-db.org/), ‟Homo sapiens” was used as the screening condition, and the outcomes were stored. After importing the file into Cytoscape v3.10.1, the degree centrality (DC), betweenness centrality (BC), and closeness centrality (CC) were determined using CytoNCA and BisoGenet. ShinyGO (https://bioinformatics.sdstate.edu/go/) web tool was used to analyze the KEGG pathways and Gene ontology. By submitting the gene symbols or the query file the result of KEGG and (GO) biological process was obtained.

## Result and discussion

### Molecular docking

Prior to molecular docking, the docking protocol was first validated by redocking the co-crystallized ligand by calculating the root-mean-square (RMS) deviation on maestro 14, which was found to be 1.99. The commonly accepted cut-off for RMS values is 2 Å, which indicates that a docking program is performing satisfactorily if it can reproduce the binding pose of a ligand within this threshold. All the 182,455 compounds were initially screened using high-throughput screening (HTVS) to study their binding affinity. This process involves docking compounds at a rate of 2 s per compound, allowing for the sorting and shortlisting of hits based on their affinity for the binding site. HTVS allows rapid screening of large compounds library, primarily used for initial rapid screening. It helps to quickly filter out compounds that are unlikely to bind effectively to target protein. However, it sacrifices some accuracy in pose prediction [47]. In contrast, following the HTVS screening, 55,872 molecules were selected for SP ligand docking. This method balances speed and accuracy, docking compounds at a rate of 10 s per compound. It provides better scoring and pose prediction by performing exhaustive sampling of ligands conformations. A cut-off dock score of − 6.5 kcal/mol was then applied to the SP docked molecules to eliminate false positives and improve the correlation between ligand poses and scores [48]. This process involves the torsion enumeration of ligands and the generation of exhaustive conformations to identify promising ligand poses. The 6.5 cut-off maximizes the separation of compounds that exhibit strong binding from weak or negligible interactions. Various studies demonstrate and validate the effectiveness of active compounds on that threshold [42]. The top molecules from SP docking (less than − 8 docking score) were then docked using the XP module to sort the best molecules based on the pose filter and desired interaction with 6NJS. This model particularly optimizes lead compounds and understands key interactions within the binding site. Typically, we chosen four compounds based on the crucial interaction and binding affinity, which is quantified through scoring functions that evaluate the strength of interaction between the ligand and target protein. As determined by their docking scores and interaction in the XP ligand docking, 5 compounds were found to exhibit hydrogen-bonding interactions with critical residue shown in Table 1.

**Table 1:** Two-dimensional interaction diagrams showing the incoming ligand (KQV) and four chosen ligands along with a synopsis of the non-bonding interactions, glide energy, and docking score

S. no	Compound name	Docking score (kcal/mol)	MM-GBSA dg-bind (kcal/mol)	Interaction diagrams	Bonding interaction	Non-bonding interactions
1	**ZINC255200499**	− 10.49	− 41.19		**H-Bond**: Ser 611, Ser 613, Arg 609, Lys 591, Ser 636, Gln 635, Ile 634, Gln 633	Glu 594, Arg 595, Ile 659, Tyr 657, Trp 623, Thr 622, Thr 620, Glu 612
2	**ZINC299817570**	− 9.20	− 54.98		**H-Bond: **Arg 609, Glu 612, Ser 611, Met 660, Glu 638, Ile 634, Glu 594, Arg 595	Ser 613, Ser 614, Pro 639, Ser 636, Gln 635, Val 637, Thr 620, Thr 622, Lys 591
3	**ZINC31167114**	− 9.15	− 36.73		**H-Bond: **Arg 609, Ser 611, Glu 612, Glu 638, Ile 634, Lys 591, Glu 594	Arg 595, Lys 591, Gln 635, Ser 636, Val 637, Pro 639, Ser 614, Thr 620, Thr 622
4	**ZINC679110988**	− 8.16	− 42.96		**H-Bond:** Ile 634, Glu 594, Tyr 657, Ser 613, Glu 612, Ser 611 **Salt- Bridge:** Arg 609	Lys 591, Arg 595, Pro 639, Glu638, Val 637, Gln 635, Ile 659, Trp 623, Thr 622, Thr 620, Ser 612
5	**KQV (Co- Crystal ligand)**	− 9.55	− 90.01		**H- Bond: **Ser 611, Glu 612, Ser 613, Glu 638, Val 637, Ser 636 **Salt Bridge: **Arg 609	Ser 614, Gln 644, Phe 610, Trp 623, Thr 620, Gln 635, Thr 657, Lys 658, Ile 659, Met 660, Leu 666

**Fig. 2:**
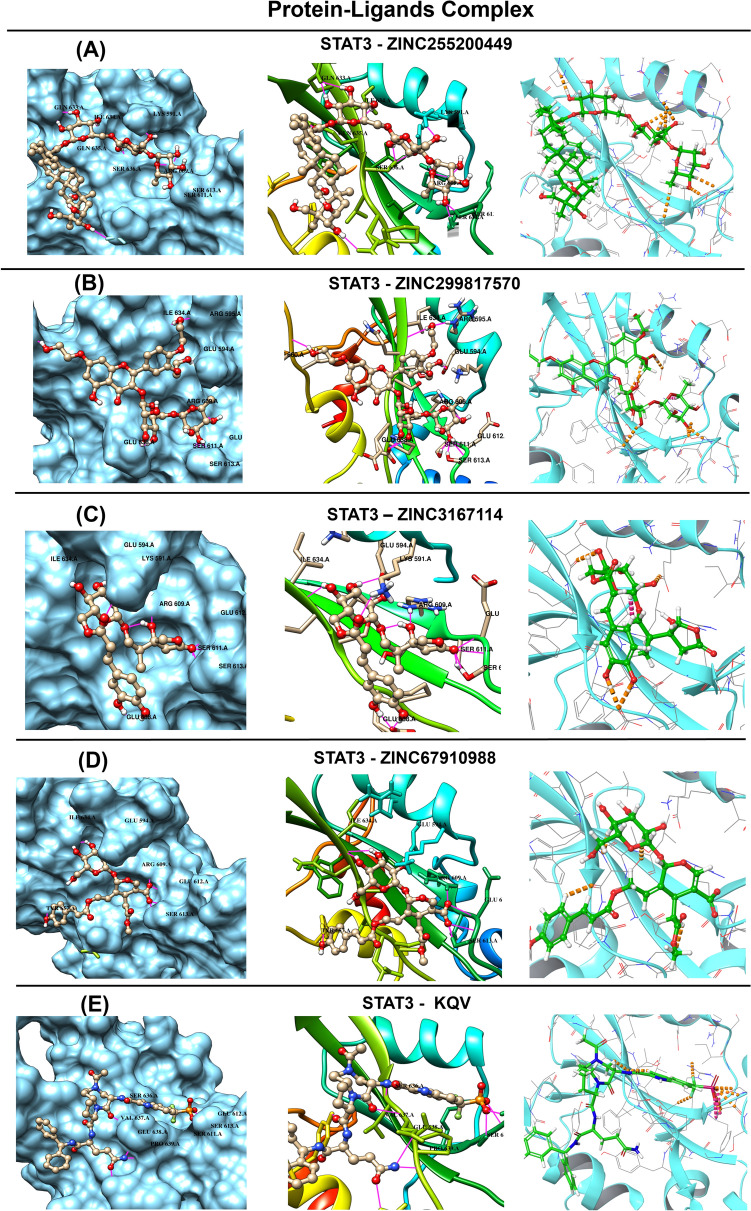
3D interaction between the protein–ligand complex. Here, figure **A** ZINC255200449, **B** ZINC299817570, **C** ZINC3167114, **D** ZINC67910988 and **E** KQV (co-crystal ligands) showing the ligand contact with the protein 6NJS after molecular docking

The critical interactions with Ser 609, Ser 611, Glu 594, Glu638, Ser 636, Ser 637, and Ser613 shown by these compounds are similar to that of the SD36 core complex of a ligand of 6NJS [19]. According to docking findings, these chemicals may occupy the STAT3 SH2 domain and impede the formation of STAT3 dimers. 3D diagram of the Protein ligand complex is illustrated in Fig. 2.

### Ligand-binding energy calculation

The stability of protein–ligand complexes formed after docking was evaluated using Prime-MM-GBSA to determine ligand binding energies. Table 1 shows that all 5 ligands exhibited stability when complexed with the protein, demonstrating binding energies exceeding − 30 kcal/mol. The co-crystallized ligand had a binding energy of − 90.01 kcal/mol. Four specific ligands (ZINC255200449, ZINC299817570, ZINC31167114, and ZINC67910988) displayed binding energies of − 41.19, − 54.98, − 36.73, and − 42.96 kcal/mol, respectively.

For the estimation of binding free energy, MM-GBSA evaluates the molecular mechanism interaction, solvation effects, and entropy changes [44]. All these parameters are crucial to maintaining the stability of the protein–ligand complex. Where lower binding free energy indicates the stronger interaction between ligand and protein. This strong interaction with protein often translates to higher therapeutic efficacy which suggests that ligand can effectively inhibit or activate the target protein. Moreover, this model also evaluates the enthalpic properties such as hydrogen bonds, van der Waals interactions, and electrostatic interactions, which stabilize the ligand–protein complex [49]. Compared to the traditional docking method, MM-GBSA provides more accurate estimation of ligand binding affinities. By calculating various conformations of ligand and its interactions with the protein, MM-GBSA helps to identify most energetically favorable binding poses [50]. Furthermore, it analyzes individual contributions from different residues in the binding site, enhancing understanding of key interactions for lead optimization. Subsequently, an ADME analysis was conducted on all ligands to assess their ADME properties after MM-GBSA.

### ADME analysis

The pharmacokinetic properties of the compounds, such as their absorption, distribution, metabolism, and excretion, were estimated using the QikProp software. Molecular descriptors were examined to evaluate drug absorption and distribution of the four compounds with the highest dock scores. These descriptors included the partition coefficient (QPlogPo/w), which fell within the acceptable range of − 2.0 to 6.5, and water solubility (QPlogS), ranging from − 6.5 to − 0.5. Table 2 displays the predicted Caco-2 cell permeability (QPPCaco), Lipinski’s rule of five compliance, and the percentage of human oral absorption. The predicted parameters for the chosen compounds were all within acceptable limits, and they adhered to Lipinski’s rule of five without any deviations. Further, the toxicity profile of all compounds was predicted, as shown in Table 3. Various parameters such as ames toxicity evaluate mutagenic potential, hepatotoxicity assesses liver damage, LD50 value quantifies acute toxicity, and hERG I and II inhibitor for prolonged QT syndrome leading to fetal ventricular arrhythmia were all compounds showing minimal or no toxicity in ADME prediction. Early in the drug development process, QikProp, a computational tool, is essential for predicting the pharmacokinetic and physicochemical characteristics of tiny organic compounds [51]. QikProp analyzes the three-dimensional molecular structure of drugs to produce a range of pertinent descriptors that evaluate the properties of absorption, distribution, metabolism, and excretion (ADME) [52]. By identifying potentially troublesome drug candidates early in the development process, this predictive capability lowers the likelihood of late-stage failures brought on by subpar pharmacokinetic characteristics [53]. QikProp specifically assesses solubility, permeability, and plasma protein binding, generating an overall ADME-compliance score that aids in prioritizing drugs with greater clinical trial success rates [54]. For the development of new drug partition coefficient (QPlogPo/w) and water solubility (QPlogS) values are important parameters, as these provide insights into compounds behavior in biological systems [55]. Where QPlogPo/w indicates the hydrophobicity of compounds, which reflect the partitions between a lipid phase and water [56]. Here, higher values indicate greater lipophilicity, which can influence absorption, distribution, and potential bioaccumulation in biological systems. Additionally, QPlogS quantifies the solubility of a compound in water, with higher values indicating better solubility, it essential for the effective formulation of the drug and delivery system [57]. These metrics also predict the compounds pharmacokinetic profile, including its ability to cross biological membranes and its overall efficacy as a therapeutic agent.

**Table 2:** ADME prediction selected four ligands and KQV using various parameters like solubility, partition, absorption, and draggability

Compounds	QPlogPo/w	QPlogS	CIQPlogS	QPlogHERG	QPPCaco	QPlogBB	Human oral absorption	Rule of five
ZINC255200449	− 2.491	− 3.195	− 5.069	− 5.741	0.445	− 6.307	1	3
ZINC67910988	− 0.488	− 2.936	− 3.821	− 4.306	0.888	− 4.401	1	3
ZINC31167114	− 1.391	− 1.633	− 3.274	− 4.317	4.5	− 3.531	1	2
ZINC299817570	− 2.423	− 2.058	− 4.666	− 6.338	0.673	− 6.042	1	3
KQV	1.023	− 5.185	− 5.734	1.534	0.011	− 5.933	1	3

**Table 3:** Toxicity profile of all compounds

Property	ZINC255200449	ZINC299817570	ZINC31167114	ZINC67910988	KQV	Unit
AMES toxicity	No	No	No	No	No	Categorical (Yes/No)
Max. tolerated dose (human)	0.018	0.261	− 0.148	0.531	0.448	Numeric (log mg/kg/day)
hERG I inhibitor	No	No	No	No	No	Categorical (Yes/No)
hERG II inhibitor	Yes	Yes	Yes	No	Yes	Categorical (Yes/No)
Oral Rat Acute Toxicity (LD50)	2.479	2.389	3.395	3.245	2.668	Numeric (mol/kg)
Oral Rat Chronic Toxicity (LOAEL)	6.658	5.437	3.928	5.228	4.338	Numeric (log mg/kg_bw/day)
Hepatotoxicity	No	No	No	No	Yes	Categorical (Yes/No)
Skin Sensitization	No	No	No	No	No	Categorical (Yes/No)
*T. pyriformis* toxicity	0.285	0.285	0.285	0.285	0.285	Numeric (log ug/L)
Minnow toxicity	12.943	6.413	3.152	4.969	9.536	Numeric (log mM)

#### Induced fit docking

Induced fit docking studies were carried out to investigate the ligand–protein binding mechanism in flexible protein structures. Each ligand was examined in 20 distinct poses in IFD. Table 4 provides a tabular description of the interactions of the ligands between the extra precision docking pose and the induced fit docking pose.

**Table 4:** Summary of the difference between XP docking and induced fit docking pose of the ligands

S no.	Drug name	XP- docking pose	IFD pose
1	ZINC255200449	**H-Bond:** Arg 609, Ser 611, Ser 613, Lys 591, Ser 636, Gln 635, Ile 634, Gln 633	Arg 609, Ser 611, Ser 613, Val 637, Ser 636, Gln 635, Ile 634, Gln 633, Arg 595, Glu 594, Met 660, Ala 662, Lys 626, Tyr 657, Glu 612, Glu 638
2	ZINC299817570	**H-Bond: **Arg 609, Ser 611, Glu 612, Met 660, Glu 638, Ile 634, Glu 594, Arg 595	Ser 613, Ser 636, Glu 638, Arg 595, Gln 633, Glu 612, Ser 611, Glu 625, Tyr 640, Lys 591, Glu 594, Lys 658, Lys 631, Arg 609, Tyr 657, Pro 639 **Pi–Cation: **Lys 591
3	ZINC31167114	**H-Bond: **Arg 609, Ser 611, Glu 612, Glu 638, Ile 634, Lys 591, Glu 594	Ser 636, Gln 635, Ile 634, Glu 594, Ser 611, Ser 613, Glu 612, Tyr 657, Val 637, Glu 638 **Pi–Pi Stacking: **Trp 623
4	ZINC67910988	**H-Bond:** Ile 634, Glu 594, Tyr 657, Ser 613, Glu 612, Ser 611 **Salt Bridge:** Arg 609	Gln 635, Ile 634, Ser 636, Lys 591, Ser 613, Arg 609, Glu 612, Ser 611, Arg 595, Glu 594, Gln 633, Tyr 657, Glu 592
5	KQV	**H- Bond: **Ser 611, Glu 612, Ser 613, Glu 638, Val 637, Ser 636 **Salt Bridge: **Arg 609	Arg 609, Ser 611, Glu 612, Ser 613, Glu 638, Ser 636, Gln 633, Thr 640, Gln 644, Gln 635, Tyr 657 **Salt Bridge: **Lys 591, Arg 609

In the case of ZINC255200449, hydrogen bonds were seen with Arg 609, Ser 611, Ser 613, Lys 591, Ser 636, Gln 635, Ile 634, and Gln 633 in the XP-docking position. On the other hand, the IFD position identified extra hydrogen bonds with Glu 638, Arg 595, Glu 594, Met 660, Ala 662, Lys 626, Tyr 657, and Glu 612. Remarkably, interactions between Tyr 657 and Glu 638 were also seen in the IFD stance that were not seen in the XP-docking pose. For ZINC299817570, hydrogen bonds were found with Arg 609, Ser 611, Glu 612, Met 660, Glu 638, Ile 634, Glu 594, and Arg 595 in the XP-docking pose and with Ser 613, Ser 636, Glu 638, Arg 595, Gln 633, Glu 612, Ser 611, Glu 625, Tyr 640, Lys 591, Glu 594, Lys 658, Lys 631, Cys 609, Tyr 657, and Pro 639 in the IFD pose. Additionally, a Pi–cation interaction with Lys 591 was unique to the XP-docking pose. In the XP-docking position, ZINC31167114 showed hydrogen bonds with Arg 609, Ser 611, Glu 612, Glu 638, Ile 634, Lys 591, and Glu 594. The interactions with Ser 636, Gln 635, Ile 634, Glu 594, Ser 611, Ser 613, Glu 612, Tyr 657, Val 637, and Glu 638 were identical in the IFD position. Interestingly, a Pi-Pi stacking interaction with Trp 623 was seen in the IFD but not in the XP-docking position. In the case of ZINC67910988, additional salt bridge interactions were found with Arg 609 and Gln 635 in the IFD stance, while the XP-docking pose revealed hydrogen bonds with Ile 634, Glu 594, Tyr 657, Ser 613, Glu 612, and Ser 611. Ile 634, Ser 636, Lys 591, Ser 613, Arg 609, Glu 612, Ser 611, Arg 595, Glu 594, Gln 633, Tyr 657, and Glu 592 were also interacted with in the IFD position. Hydrogen bonds were formed with Ser 611, Glu 612, Ser 613, Glu 638, Val 637, and Ser 636 in the XP-docking position in the case of KQV. Furthermore, interactions between Arg 609 and salt bridges were observed. Interactions with Arg 609, Ser 611, Glu 612, Ser 613, Glu 638, Ser 636, Gln 633, Thr 640, Gln 644, Gln 635, and Tyr 657 were noted in the IFD posture. Salt bridge interactions involving Lys 591 and Arg 609 were observed in IFD which were absent in the XP-docking position. Mostly residue Ser 614, Gln 644, Phe 610, Trp 623, Thr 620, Thr 657, Lys 658, Ile 659, Met 660, and Leu 666 are shown flexibility.

### WaterMap analysis for hydration site prediction

The process of water map analysis uses thermodynamic molecular dynamics simulation to determine the enthalpy and entropy of each hydration site in relation to the bulk solvent. The theory of Lazaridis and Karplus, which takes into account the substantial contributions of solvent reorganization energy and entropy to the solvation-free energy in an inhomogeneous system, is taken into consideration in the WaterMap study. Red spheres on WaterMap indicate high-energy hydration sites that are easily replaceable. This makes it possible to determine whether hydration sites are present. Green spheres firmly packed around the ligand are the ones with less energy. A comprehensive method was used to ascertain the quantitative relationship between the hit compounds' binding affinities and the calculated hydration site energetics. WaterMap analysis provides significant insights into the hydration sites, enhancing our understanding of how water molecules influence ligand binding affinity [58]. Using a structure-based approach, WaterMap analyzes the positions and energetics of water molecules in binding pockets. By revealing the site of hydration, it can stabilize ligand interactions through enthalpic and entropic properties. This approach demonstrates that water molecules occupy critical positions that either facilitate or hinder ligand binding [59]. These specific sites of hydration are identified as key to optimizing ligands. For instance, WaterMap has shown that certain water molecules can be displaced by ligands, which suggests favorable changes in binding affinity. This understanding helps to design effective compounds in drug discovery [60]. Analysis of the apoprotein area (around 5Ǻ of the ligand binding pocket) revealed the presence of 38 water molecules. Water molecules with hydration energies between − 1.77 and 2.46 kcal/mol and occupancy values between 0.84 and 0.28 were found in the ligand binding pocket, demonstrating the vast range of water molecules surrounding the pocket, as shown in Table 5

**Table 5:** Predicted site of hydration, occupancy, and thermodynamic energy of the available hydration sites within five angstroms selected ligands

Site	Occupancy	ΔH (kcal/mol)	TΔS (kcal/mol)	ΔG (kcal/mol)
7	0.84	− 4	3.5	− 0.5
11	0.79	− 1.45	2.19	0.74
15	0.75	− 0.52	1.79	1.27
16	0.75	− 0.14	1.66	1.52
19	0.73	− 2.4	2.09	− 0.31
25	0.66	− 0.87	2.07	1.2
27	0.65	0.63	1.47	2.1
31	0.62	0.91	1.55	2.46
32	0.62	− 1.85	1.59	− 0.26
36	0.58	− 1.23	1.17	− 0.06
38	0.57	− 1.4	1.81	0.41
41	0.54	1.11	1.35	2.46
43	0.54	− 2.55	1.28	− 1.27
48	0.49	0.09	1.02	1.11
54	0.46	0.09	0.94	1.03
59	0.45	− 0.56	0.93	0.37
66	0.42	0.13	0.83	0.96
67	0.42	− 0.37	0.78	0.41
68	0.41	0.4	0.81	1.21
71	0.4	− 2.63	0.86	− 1.77
73	0.4	− 0.51	0.77	0.26
78	0.39	− 0.56	0.73	0.17
80	0.37	− 1.23	0.64	− 0.59
81	0.37	− 0.28	0.72	0.44
84	0.36	− 0.55	0.65	0.1
88	0.35	0.36	0.63	0.99
94	0.33	− 2.08	0.69	− 1.39
95	0.32	0.4	0.55	0.95
98	0.32	− 0.65	0.52	− 0.13
104	0.31	1.69	0.48	2.17
109	0.3	0.08	0.52	0.6
121	0.29	− 0.51	0.45	− 0.06
122	0.28	0.72	0.46	1.18
123	0.28	− 0.8	0.48	− 0.32
127	0.28	− 0.1	0.85	0.75
128	0.28	0.16	0.5	0.66
129	0.28	− 0.29	0.43	0.14
132	0.28	− 0.5	0.49	− 0.01

It was found that every ligand covered the hydrophobic pockets that the hydration sites 11, 32, 73, 81, and 94 created. Hydration sites 71, 94, 43, 80, and 7 are in the protein’s hydrophilic pocket; their lower negative energy indicates strong binding. Table 6 lists the potential hydration sites within 5 angstroms of the top ligand that was chosen in the docking site of the STAT3 SH2 domain, along with the projected overlaps greater than 0.4. Compound ZINC255200449 contains 16 water molecules with ligand relative free energy between 2.17 and − 0.31 kcal/mol. Similarly, 23 water molecules were present around the ligand ZINC299817570 with occupancy in the range of 0.28 to 0.84 kcal/mol and ΔG with free energy from 2.46 to − 1.77 kcal/mol. Compound ZINC31167114 contained 11 hydration sites with free energy ranging from 1.18 to − 1.77 kcal/mol. Compound ZINC67910988 had 15 hydration sites with free energy ranging from 1.52 to − 1.39 kcal/mol. Co-crystal compound (KQV) contains 18 sites of hydration with free energy ranging from 2.46 to − 1.77 kcal/mol.

**Table 6:** Estimated site of hydration and overlaps of the selected ligand in the docking site of 6NJS

SN	Name	Site and overlap	Diagram
1	ZINC255200449	11(1), 19(1), 25(1), 27(0.64), 32(1), 73(1), 78(1), 81(1), 88(1), 95(1), 121(1), 122(1), 129(0.69)	
2	ZINC299817570	7(1), 11(1), 16(1), 19(1), 25(0.83), 32(1), (38(0.4), 41(0.63), 48(1), 54(1), 66(0.4), 67(0.52), 71(1), 73(0.64), 81(1), 88(0.93), 94(0.87), 104(1), 109(1), 127(0.42), 128(1), 129(1), 132(0.51)	
3	ZINC31167114	7(1), 11(0.91), 19(1), 32(1), 38(1), 54(0.68), 71(1), 73(0.94), 81(1), 94(1), 122(1)	
4	ZINC67910988	7(1), 11(1), 15(1), 16(1), 32(1), 36(1), 38(0.52), 43(1), 66(0.47), 73(1), 80(1), 81(1), 94(1), 122(1), 132(0.58)	
5	KQV	11(1), 15(1), 19(0.6), 31(1), 32(1), 36(1), 41(0.97), 54(1), 59(1), 66(1), 68(1), 71(1), 80(1), 94(1), 98(1), 104(1), 123(1), 128(1)	

### Molecular dynamics simulation

#### RMSD analysis

To investigate protein–ligand interactions, molecular dynamics (MD) simulations were conducted on selected compounds to analyze the atomic and molecular movements of the ligand-receptor complex under physiological conditions. To assess the stability of compounds, two metrics include Root Mean Square Deviation (RMSD), which measures the average deviation of atomic positions from their initial configuration and occupancy of hydrogen bonds, it quantifies the frequency with which hydrogen bonds form between donor and acceptor atoms over the course of the simulation. The MD simulation analysis between ZINC67910988 and STAT3, the RMSD plot indicated a stable ligand RMSD throughout the entire 500-ns simulation, ranging from 2 to 3.4 Ǻ. The protein also exhibited stable RMSD values during the simulation period, varying from 2.5 to 4.8 Ǻ. Direct hydrogen-bonding interactions were observed between the amino acid residue Glu 594 and the hydroxyl group (accounting for 96% and 85% of the time). Additionally, Arg 609, Ser 611, Glu 612, and Ser 636 formed direct hydrogen bonds with the carboxyl group of the ligand (accounting for 40%, 42%, 44%, 51%, and 67% of the time, respectively). The MD simulation revealed a loss of interactions with residues Ile 634, Tyr 657, and Ser 613, which were previously present in XP docking.

In the MD simulation analysis of the ligand–protein interaction between ZINC31167114 and STAT3, the protein RMSD plot remained stable throughout, ranging from 3.2 to 5 Ǻ, while the ligand plot exhibited minor fluctuations at 20 ns, 270 ns; however, after 350 ns RMSD value raise up to 5 Ǻ. Subsequently, the ligand demonstrated good stability with the protein up to 500 ns, with RMSD values between 2.4 and 6 Ǻ. Strong, direct hydrogen-bonding interactions were observed between the hydroxyl group and amino acid residues Glu 594, Glu 638, and Val 637 (accounting for 72%, 62%, 56%, and 34%). Glu 636 and Glu 612 also formed direct hydrogen bonds with the carboxyl group (accounting for 49% and 59%). Moreover, Glu 612, Ser 613, and Ser 636 established direct hydrogen bonds with the carboxyl group in the ligand. During the MD simulation, interactions with residues Arg 609, Ser 611, Lys 591, and Ile 634, which were present in XP docking, were lost. Conversely, new hydrogen bonds formed with residues Val 637 and Arg 595 during the simulation.

In the MD simulation analysis of the ligand–protein interaction between ZINC255200449 and STAT3, the protein RMSD plot remained stable at 220 ns. After that major fluctuations were observed, and RMSD raise up to 30 Ǻ. However, fluctuation ligands still show stability with protein till 500 ns. The protein also exhibited a stable RMSD during the simulation period, ranging from 2.4 to 4.2 Ǻ. Hydrogen bond interaction was observed with the residue Glu 594 and Ser 636, accounting for 37%, 33%, and 36% of the time. During the MD simulation, interactions with residues Ser 611, Ser 613, Lys 591, Glu 635, and Gln 633, which were present in XP docking, were lost. Conversely, new hydrogen bonds formed with residues Glu 594 during the simulation.

In the analysis of the interaction between ZINC299817570 and STAT3, the protein RMSD plot remained consistent throughout, fluctuating between 2 and 4 Ǻ, while the ligand plot exhibited stability up to 190 ns after that, showing a drift reaching 18 Ǻ RMSD till 500 ns. Direct hydrogen bonds were formed between the hydroxyl group and residues Glu 612 and Ser 636, accounting for 65% and 47%, respectively. Likewise, Lys 591 formed direct hydrogen bonds with both carboxyl and hydroxyl groups, accounting for 42% and 46% at 200 ns. While 500-ns MD simulation revealed a loss of hydrogen bond interactions with all residues which were previously observed in XP docking. Although compounds ZINC255200449 and ZINC299817570 both shown good stability and interaction during shorter simulation (200 ns). As the time of simulation extends over longer, the system might be exploring broader conformational space, structural flexibility, and environmental interactions, which can increase sampling lead to discovering the less stable conformations that may not have been populated during shorter simulations, resulting in a loss of stability and potential disruption of hydrogen bonds [61]. Fluctuations in temperature and solvation over longer simulations also alter the hydrogen bonds intensity and strength [62].

**Fig. 3:**
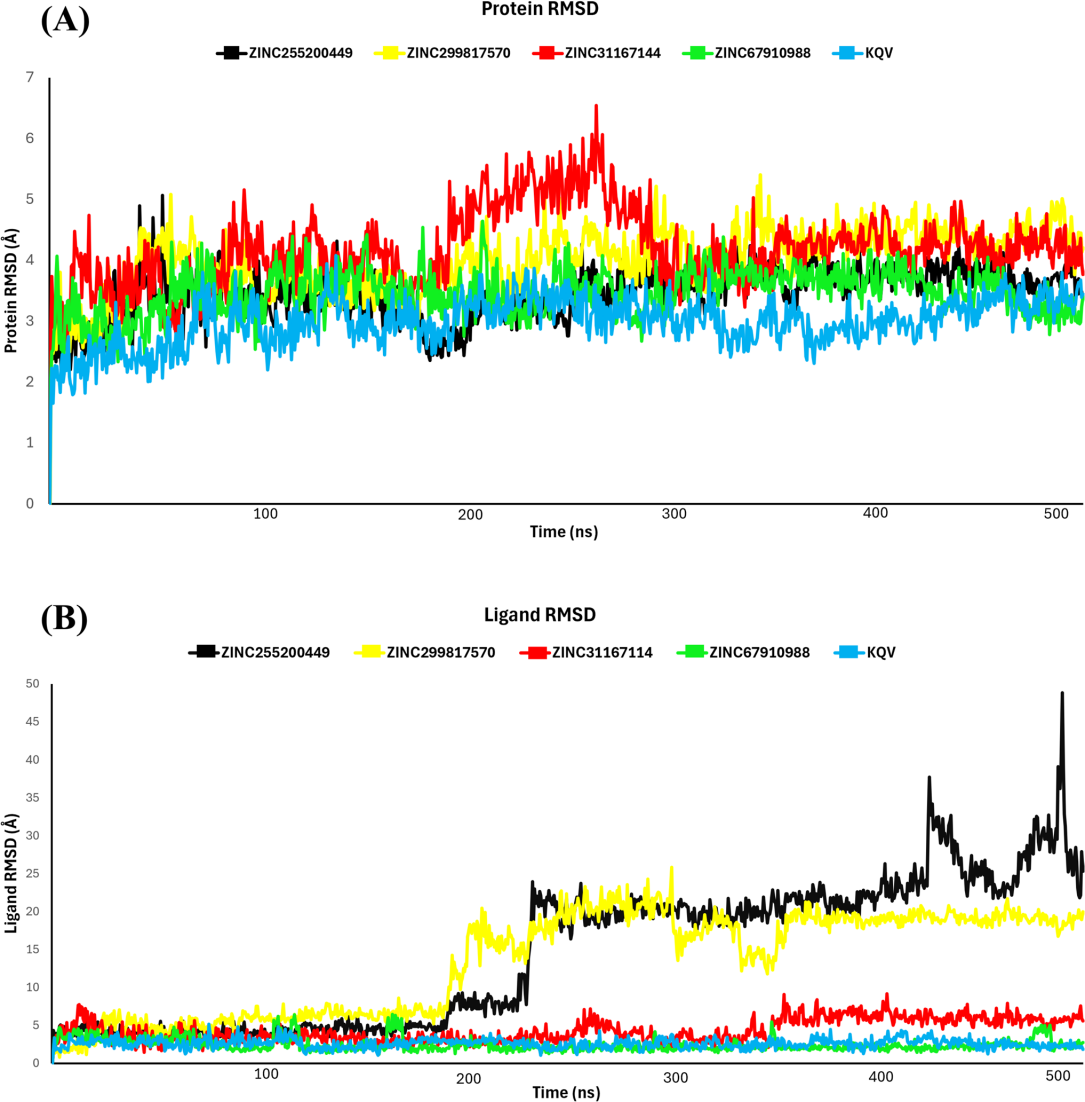
**A** RMSD of the five ligands in complex with protein backbone at 500-ns MD simulations. **B** RMSD of the protein with five ligands at 500-ns MD simulations

Furthermore, an analysis of the interaction between KQV and STAT3 was performed. The root-mean-square deviation (RMSD) plot for the protein remained stable throughout the simulation, fluctuating between 2 and 3.5 Å, while the ligand exhibited a consistent RMSD of 4 Å. This stability was observed for more than 30% of the simulation time during the 500 ns simulation, RMSD plot of all compounds depicted in Fig. 3. Overall, all compounds has been binds with pY binding pocket of STAT3 and form interaction with curial residues which responsible in STAT3 activation [15].

#### Hydrogen bond analysis

**Fig. 4:**
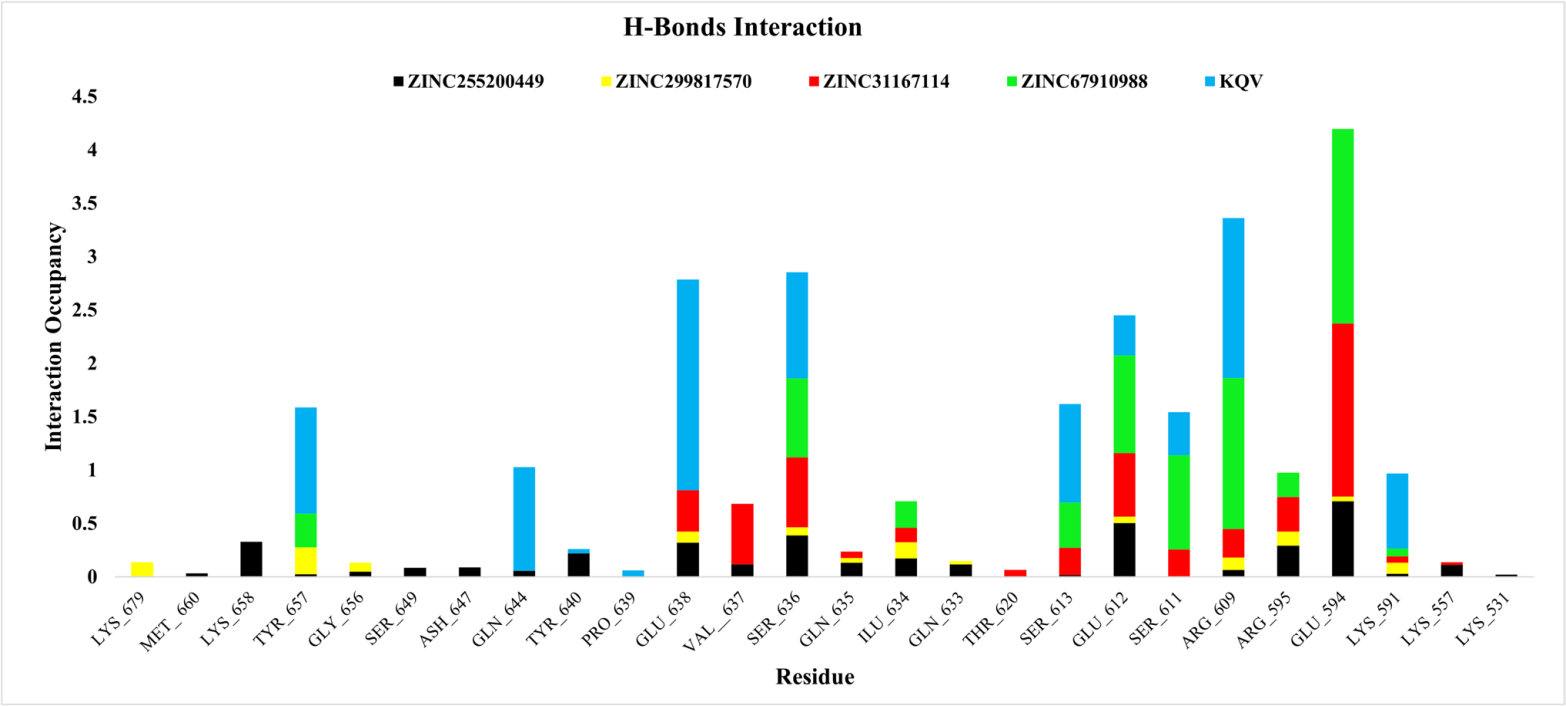
Hydrogen bond occupancy percentage of all residues which are showing contact with protein

Hydrogen bonds are a crucial factor in molecular dynamics (MD) simulations as significant influence on molecular interactions and stability within the biological and chemical systems [63]. These bonds play a pivotal role in the maintenance of the structural integrity and stability of protein–ligand complexes, which is essential for accurate modeling in computational study. Hydrogen bond analysis allows researchers to track their formation and duration throughout the simulation, providing insights into the dynamics and interactions [64]. Moreover, understanding hydrogen bonding in MD simulation can help elucidate complex phenomena, such as hydration effects and the formation of stable structures in solution, thereby enhancing the predictive potency of simulations. Hydrogen bonds with critical residues such as R609 and S613 within the pY + 0 pocket of the SH2 domain [15]. This interaction is essential for the binding affinity such as pervious inhibitors CJ-887, which mimics pY705 by utilizing a phosphorylated phenol group that engages these residues through hydrogen bonding [15]. The protein–ligand interaction shows how long the ligand and protein amino acids were in touch over the course of the simulation. A score of six means that 60% of the 500 ns of simulation time was occupied. In ZINC255200449, the residues Met 660, Lys 658, Tyr 657, Gly 656, Ser 649, Asp 647, Gln 644, Tyr 640, Glu 638, Val 637, Ser 636, Gln 635, Ile 634, Gln 633, Ser 613, Glu 612, Arg 609, Arg 595, Glu 594, Lys 591, Lys 557, and Lys 531 showed occupancy of 0.032, 0.328, 0.024, 0.048, 0.086, 0.089, 0.058, 0.221, 0.32, 0.118, 0.387, 0.131, 0.172, 0.117, 0.016, 0.502, 0.066, 0.29, 0.707, 0.028, 0.111, and 0.019, respectively. In ZINC299817570, the residues Lys 679, Tyr 657, Gly 656, Glu 638, Ser 636, Gln 635, Ilu 634, Gln 633, Glu 612, Arg 609, Arg 595, Glu 594, and Lys 591 showed occupancy of 0.136, 0.25, 0.084, 0.104, 0.077, 0.045, 0.152, 0.032, 0.06, 0.114, 0.132, 0.044, and 0.103, respectively. In the ZINC31167114, the residues Glu 638, Val 637, Ser 636, Gln 635, Ile 634, Thr 620, Ser 613, Glu 612, Ser 611, Arg 609, Arg 595, Glu 594, Lys 591, and Lys 557 showed occupancy of 0.388, 0.566, 0.656, 0.058, 0.135, 0.063, 0.256, 0.598, 0.257, 0.269, 0.326, 1.622, 0.063, and 0.027. In the ZINC67910988, the residues Tyr 657, Ser 636, Ile 634, Ser 613, Glu 612, Ser 611, Arg 609, Arg 595, Glu 594, and Lys 591 had occupancy scores of 0.316, 0.737, 0.248, 0.422, 0.915, 0.881, 1.412, 0.227, 1.823, and 0.07, respectively. In KQV, residues Tyr 657, Gln 644, Tyr 640, Pro 639, Glu 638, Ser 636, Ser 613, Glu 612, Ser 611, Arg 609, and Lys 591 showed occupancy of 0.998, 0.969, 0.037, 0.061, 1.973, 0.996, 0.926, 0.373, 0.405, 1.499, and 0.703 respectively. Compound ZINC67910988 showed better hydrogen bonds occupancy than the rest of the compounds. Occupancy of compound ZINC67910988 was higher as compared to other compound and similar to that of KQV (Co-Crystal ligand) Bar protein–ligand graph of hydrogen bonds occupancy are shown in Fig. 4.

**Fig. 5:**
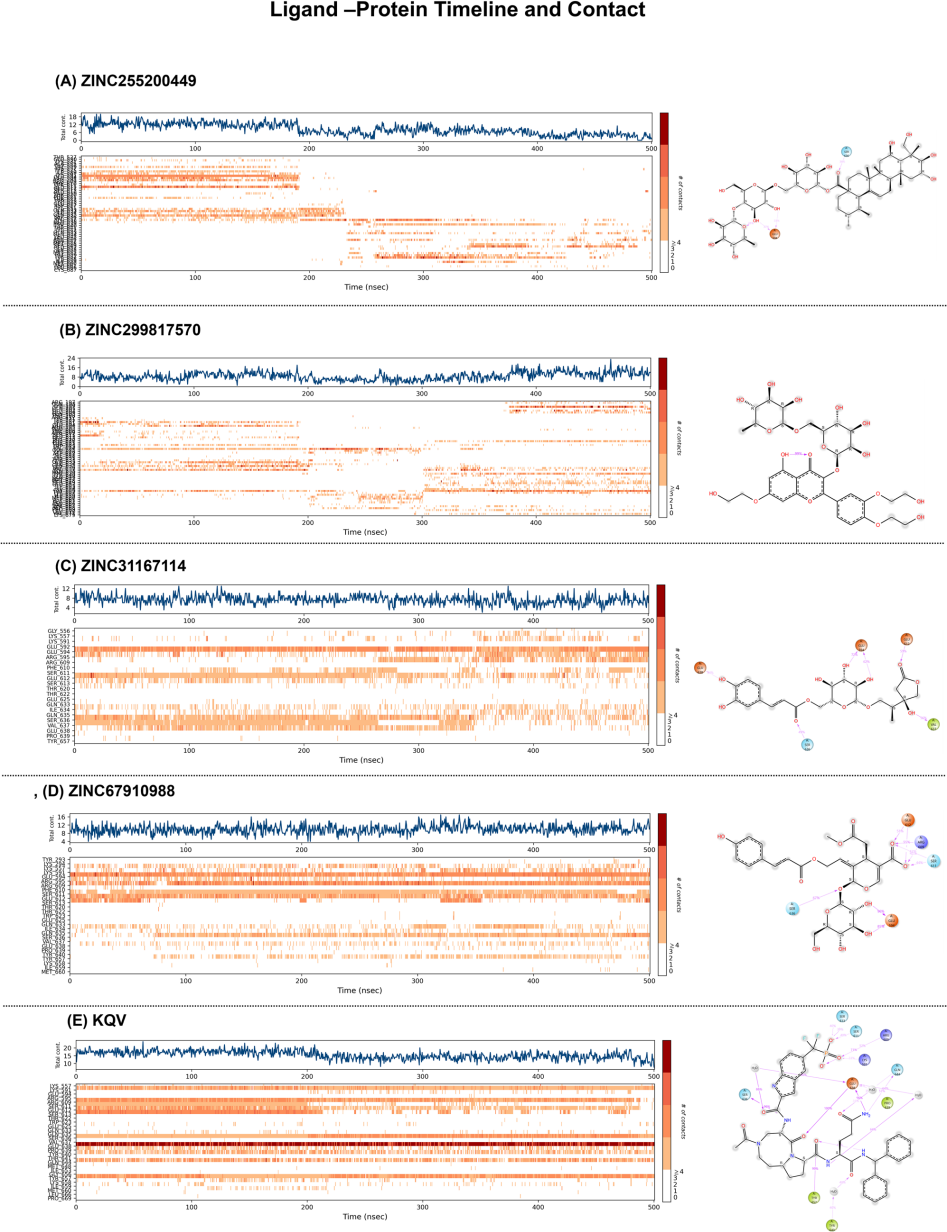
Time line chart of protein—ligands contact for all compunds **A** ZINC255200449, **B** ZINC299817570, **C** ZINC31167114, **D** ZINC67910988, and **E** KQV

The protein–ligand hydrogen contacts histogram and timeline of selected compounds (ZINC255200449, ZINC299817570, ZINC31167114, ZINC67910988) and co-crystallized ligand (KQV) are shown in Fig. 5.

#### Thermal MM-GBSA

**Fig. 6:**
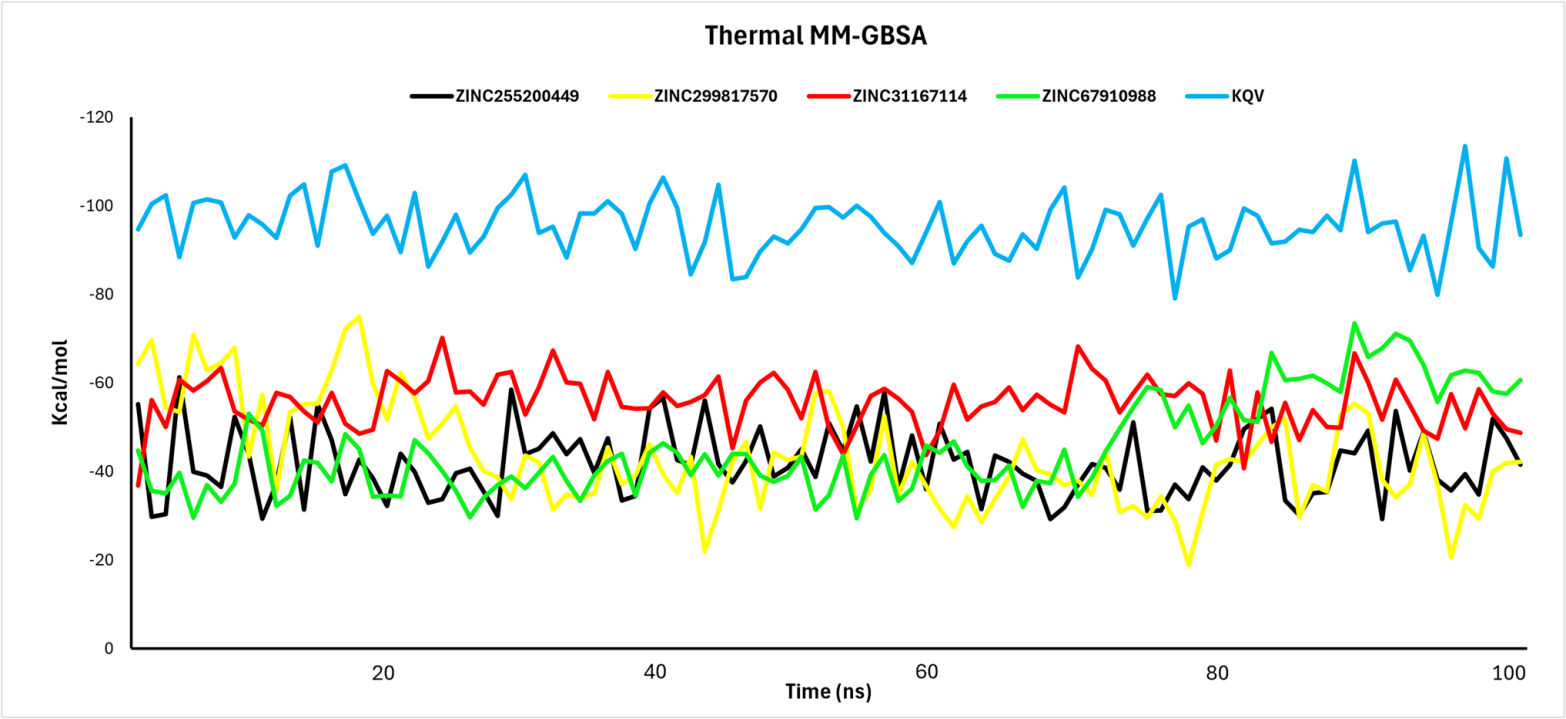
Ligand-binding energy of all four compounds and KQV obtained from every 10th frame of 1002 frames after running MD simulation

Following molecular dynamics (MD) studies, thermal MMGB-SA was conducted on the ligand–protein complexes to assess binding free energy stability. Calculations were made at every 10th frame across 1000 frames. For STAT3, ZINC255200449 dG bind ranged from − 61.21 to − 29.21 kcal/mol. ZINC299817570 exhibited values between − 74.97 and − 18.92 kcal/mol. ZINC31167114 binding energy range from − 70.12 to − 36.78 kcal/mol, while ZINC67910988 dG binding energy ranged between − 73.44 and − 29.48 kcal/mol. The co-crystal ligand KQV displayed binding energy between − 113.43 and − 79.07 kcal/mol. Figure 6 illustrates the thermal MMGB-SA results graphically. Although the binding energy range for all molecules exceeds that of the co-crystal ligand, each molecule demonstrated values within the -30 kcal/mol, which is considered acceptable for ligand stability.

## DFT analysis

**Fig. 7:**
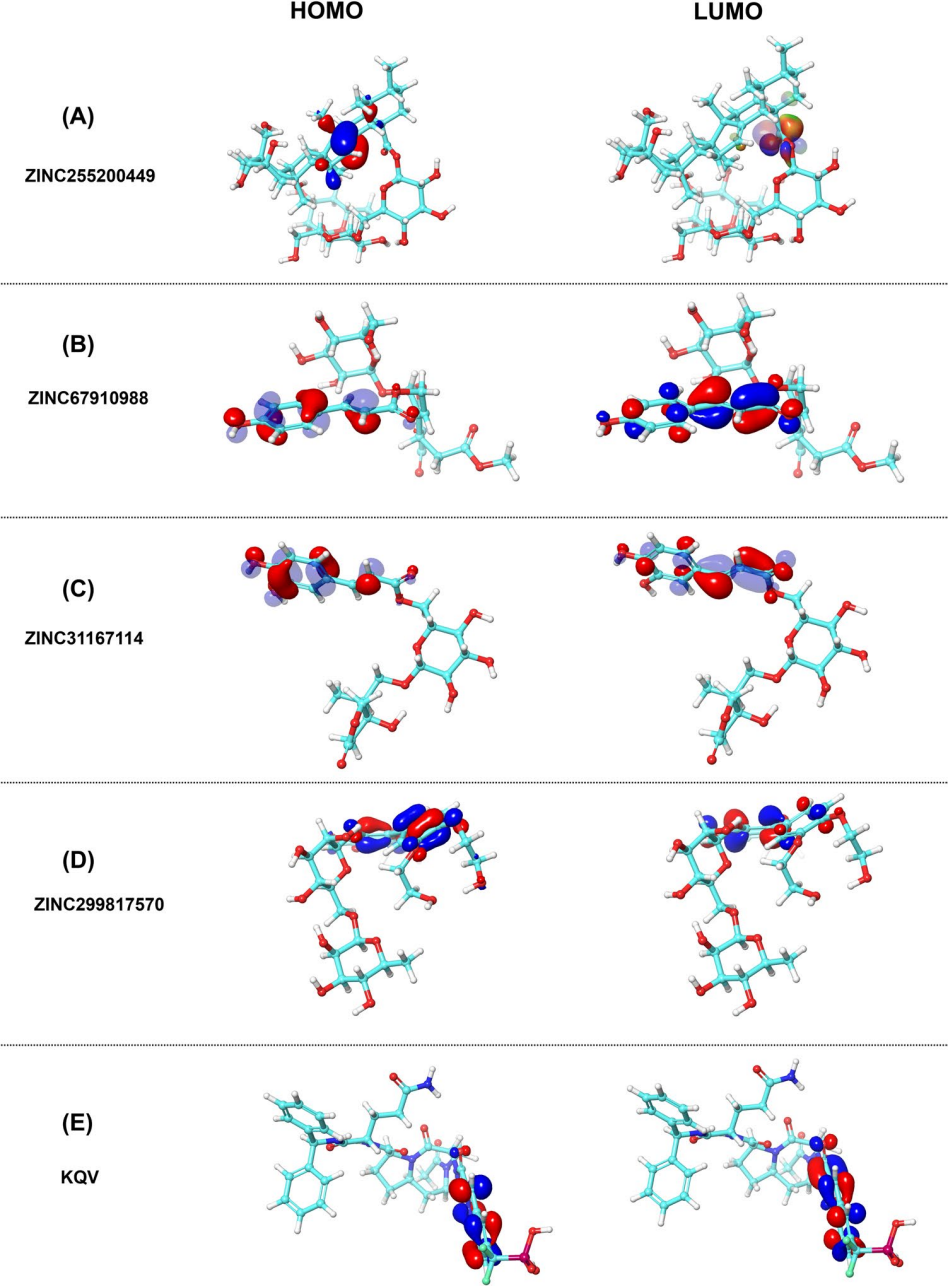
HOMO–LUMO orbitals of all compounds determined using DFT method

To determine energetic state and electronic properties at atomic level DFT method was performed for electronic structure calculation. The presence of HOMO and LUMO sites here indicates the importance of frontier orbitals in depicting compound properties (electron donor and acceptor, charge transfer). The DFT results shown HOMO to LUMO ZINC255200449; − 0.223 eV to − 0.0154 eV, ZINC67910988; − 0.223 eV to 0.678 eV, ZINC31167114; − 0.214 eV to − 0.0676 eV, ZINC299817570; − 0.217 eV to − 0.0740 eV, and KQV; and − 0.224 eV to − 0.050 eV and Solvation energy (− 68.7, − 93.4, − 38.82, − 43.52, and − 69.43 kcal/mol). Score for all compounds is displayed in Table 7. The HOMO results are directly proportional to ionization potential, while LUMO indicates the electron affinity. The HOMO and LUMO sites for all compounds are shown in Fig. 7. Here, the HOMO and LUMO-associated energy scores display the delicate behavior of bound electrons of both compounds. All compounds show almost the same (± 5) HOMO Eigenvalue which indicates rigid binding of electrons with the nuclei and lower LUMO Eigen score shows stronger electron affinity. In terms of kinetic and chemical energies, the HLG (HOMO–LUMO energy gap) shows how stable a compound is; compounds with a smaller HLG exhibit higher activity. These findings provide more evidence in favor of the anticipated atom-based 3D-QSAR model. The compound with the highest solvation energy has a high negative score than lowest solvation energy, which indicate that it is more soluble in water. Compounds calculated solvation energy can be utilized for pharmacokinetic optimization research.

**Table 7:** Parameters and values of DFT

Compounds	HOMO (eV)	LUMO (eV)	HLG (eV)	Solvation energy (kcal/mol)
ZINC255200449	− 0.22369	− 0.015423	− 0.20826	− 68.7
ZINC67910988	− 0.22365	− 0.067845	− 0.1558	− 93.4
ZINC31167114	− 0.21471	− 0.067628	− 0.14708	− 38.82
ZINC299817570	− 0.21726	− 0.074037	− 0.14322	− 43.52
KQV	− 0.2248	− 0.050823	− 0.17398	− 69.43

## Identification of multitargets and pathways of compounds by network pharmacology

**Fig. 8:**
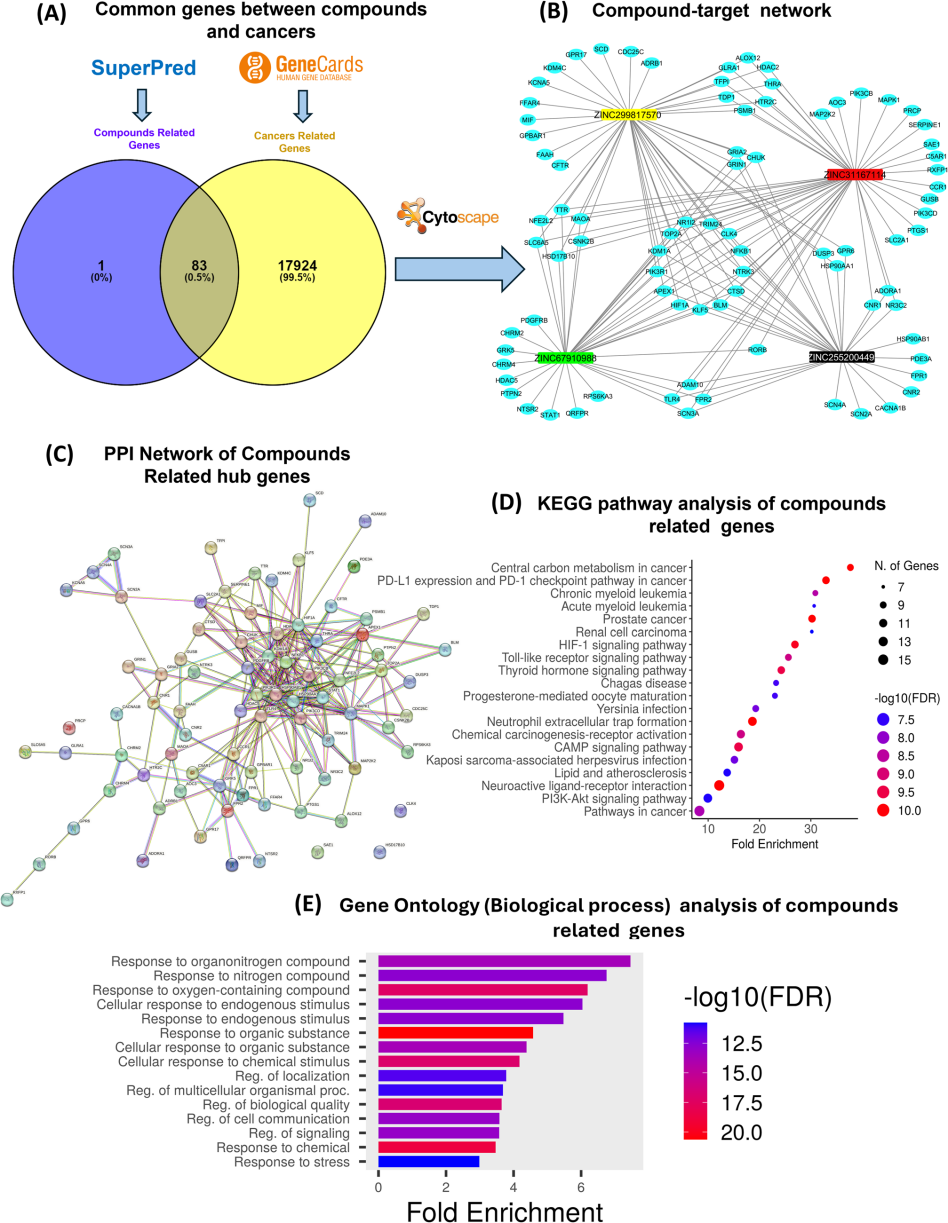
Network Pharmacology-based prediction of compound, with multi-cancer target. **A** Venny diagram. **B** Network diagram of compounds and their targets. **C** PPI network of 83 compound-related genes. **D** KEGG pathway analysis. **E** Gene ontology

For multitarget and pathway analysis of compounds via network pharmacology, 84 targets related to the compounds were obtained from the Superpred, and 18,007 cancer-related targets were obtained from the Genecard databases. Venn analysis showed that there were 83 overlapping targets between compounds and cancer-related targets, as shown in Fig. 8A. To elucidate the relationship between compounds and potential cancer targets, a compound target network was constructed using Cytoscape 3.10.0, consisting of 87 nodes and 161 edges, as shown in Fig. 8B, with a network density of 0.043 and a network diameter of 4. The detailed information of this network is shown in Table 8. The created network revealed that the compounds ZINC255200449 (degree = 32), ZINC299817570 (degree = 44), ZINC31167114 (degree = 48), and ZINC67910988 (degree = 37) exhibited varying degrees of connectivity. Were ZINC31167114 showing 48 degree, compounds with higher degrees in the network are significant due to their enhanced connectivity and influence on biological systems. In network the degree of node refers to the number of connections it has to other nodes, which can indicate its importance in mediating interactions within biological processes. This serves as key players in disease progression or drug response, making them valuable targets for network-based pharmacology approaches. 12 targets (NR1I2, TRIM24, CLK4, NFKB1, NTRK3, CTSD, BLM, KLF5, HIF1A, APEX1, PIK3R1, KDM1A, and TOP2A) in the network showed interaction with all four compounds. All these targets showed relationship with cancer like Pregnane X receptor (PXR), or NR1I2, is a transcription factor that controls genes related to pharmacokinetics, chemotherapy resistance, and the advancement of cancer [65]. Tripartite Motif-containing protein 24 (TRIM24) is an E3 ligase that ubiquitinates p53. Numerous studies demonstrate that abnormal expression of human TRIM24 is linked with various tumors and cellular dysregulation [66]. CLK4 is a member of the Cdc2-like kinase (CLK4) family, targeting CLK4 inhibits the metastasis and progression of breast cancer [67]. Nuclear factor kappa B subunit 1, or NFκB1, mediates immunological response and is the connecting link between inflammation and cancer [68]. Cell survival is regulated by NTRK3, a member of the neurotrophin receptor family. Considering its role as a dependency receptor, it may function as a tumor suppressor gene or an oncogene [69]. Cathepsin D, or CTSD, is a lysosomal aspartyl protease. That plays a crucial role in invasion and metastasis [70]. Homozygous mutations of the BLM gene are the cause of different malignancies, including breast cancer [71]. KLF5, a fundamental transcriptional factor, is well expressed in epithelial cells and can promote and repress tumor growth [72]. Numerous physiological processes are regulated by HIF1α, including the regulation of hematopoietic stem cells, angiogenesis, glucose metabolism, apoptosis, cell proliferation, survival, and immune cell activation [73]. Aberrant expression of apurinic-apyrimidinic endonuclease–1 (APEX1) has been reported in numerous human solid tumors and is positively correlated with cancer progression [74]. The regulatory subunit p85 is encoded by PIK3R1, an additional component of the PI3K pathway. Its mutations are not as common as the loss of PTEN function in lung cancer or the PIK3CA mutation [75]. A FAD-dependent H3K4me1/2 and H3K9me1/2 demethylase, lysine-specific histone demethylase 1A (KDM1A, often referred to as LSD1), was discovered to be increased in a number of malignancies and was associated with poor prognosis [76]. Important nuclear protein topoisomerase II alpha (TOP2A) is highly expressed in cells that are proliferating and growing and is present in many kinds of cancer [77]. Overall, all compound-related hub genes were found to be associated with cancers. To understand complex interaction between all overlapped 83 genes, a PPI network was constructed. This network contained 83 nodes and 239 edges in STRING with medium confidence (0.400) as shown in Fig. 8C.

**Table 8:** Parameters and values of compounds-targets cytoscape network

Parameters of network	Values
Number of edges	161
Number of nodes	87
Network density	0.043
Network diameter	4
Network radius	2
Average number of neighbors	0.527
Network heterogeneity	2.217
Characteristic path length	2.587
Network centralization	0.527

PPI network of 4 compounds and cancer-related 83 genes interact with each other by 239 edges. It suggests that it might interact with several biological pathways and metabolic pathways, which are essential for tumor development and survival, as well as other important processes implicated in the progression of cancer [78, 79]. The metabolic flexibility of cancer cells may be disrupted, for example, by their influence on energy production and lipid metabolism [80]. These compounds may also influence signal transduction pathways, which are frequently dysregulated in cancer and impact gene expression and cellular communication. Targeting several nodes in these interrelated pathways allows the compounds to work in concert to suppress tumor growth, encourage apoptosis, and strengthen the immune system defenses against cancer cells, all of which improve clinical outcomes.

## KEGG pathway enrichment analysis

To further investigate the KEGG Pathway analysis, the 83 overlapped target genes screened in PPI network were analyzed by enrichment and KEGG pathway. In total, there were 143 KEGG pathway enrichment results. The results indicated that the following pathways contain a higher number of genes: (hsa05200) pathways in cancer, (hsa04151) the PI3K-Akt pathway, (hsa04080) neuroactive receptor activation, (hsa05235) PD-L1 expression and PD-1 checkpoint pathway in cancer, and (hsa04024) cAMP signaling pathways. The first 20 representative signaling pathways in the KEGG bubble chart is shown in Fig. 8D. To further investigate Gene ontology (biological process) of 83 overlapped genes to understand functions and biological pathways of genes product. The results showed that GO biological processes were related to compounds genes and included response to organonitrogen compounds (GO:0010243), response to organic cyclic compounds (GO:0014070), response to external stimulus (GO:0032101), regulation of locomotion (GO:0040012), and response to oxygen-containing compounds (GO:1901700). Bar plot of GO enrichment score is shown in Fig. 8E. The organonitrogen compounds regulate cellular functions, such as locomotion, secretion, and gene expression, in response to nitrogen stimuli [81]. Organic cyclic compounds facilitate the modulation of metabolic pathways and signaling processes due to their association with cyclic structures in biological systems where they interact [82]. The ability to respond to external stimuli demonstrates that an organism can adapt, a key component of survival [83]. The regulation of locomotion is a behavioral process that suggests certain genes, whose expression fluctuates in response to these compounds, may play a fundamental role in these mechanisms. Furthermore, the enrichment of oxygen-containing compounds indicates potential effects on oxidative stress responses and metabolic pathways linked to aerobic respiration and energy metabolism [84].

## Conclusion

STAT3 is a critical transcription factor that regulates the growth, survival, and differentiation of many cells. Deregulated STAT3 activation has been directly linked to many human cancers, highlighting it as a potential target for cancer therapy. Using an in silico method, the phytochemical library from the ZINC database was screened for affinity to the protein STAT3 SH2 domain in the current work. Through docking, a powerful molecule with a high affinity for STAT3 was discovered. The drug-like characteristic of the hit molecules was discovered using a QikProp study of the pharmacokinetic properties of the chosen compounds. The kind and function of the hydration sites around the ligand-enzyme complexes were also determined by WaterMap analysis of the compounds that were shortlisted. The chosen compounds’ molecular dynamics simulation demonstrates stability toward the target. Ultimately, four powerful compounds with strong enzyme-binding properties were discovered. However, molecular dynamics simulation studies revealed that ZINC67910988 was the most stable of the chosen compounds. The stability of ZINC67910988 in MD simulation is nearly identical to co-crystal ligand KQV; nevertheless, the docking score is higher than that of KQV. SD36 is a small-molecule degrader of STAT3 that exhibits a strong anticancer effect against a range of malignancies. According to certain preclinical reports, SD36 has demonstrated dose-limiting toxicity. According to various preclinical findings, SD36 may cause hepatotoxicity [85], and it has little to no effect on (Colorectal Cancer) CRC cell proliferation [86]. SD36 limited solubility causes substantial challenges to both its bioavailability and therapeutic efficacy. Additionally, available STAT3 inhibitors such as OPB31121, WP1066, and stattic are suffering poor therapeutic efficacy and serious toxic effects in clinical and preclinical studies [87]. ZINC67910988 is a natural product (Jaslanceoside B) derived from plant sources, according to TargetMol (https://www.targetmol.com/compound/jaslanceoside%20b). It is a secoiridoid glucoside that have been isolated from the stems and leaves of J. lanceolarium. Jasminum species have been little studied for their biological activity. Only few literatures have been available, and antiviral effects against hepatitis B have been reported collected extract from J. grandiflorum [88]. Antimicrobial, antifungal, antioxidative, and anti-inflammatory activity of Jasminum species have been reported in various studies [89]. These findings suggest compound ZINC67910988 might be a strong candidate for further investigations. Further network pharmacology was performed, which provided a broader understanding of compounds by identifying the key biological pathways and multiple targets that a compound might be interacting with. This could help enhance these compounds' therapeutic efficacy and play a crucial role in minimizing off-target effects for further preclinical and clinical studies. However, computational techniques provide valuable insights into binding affinities, stability, and pharmacokinetic profiles but do not fully replicate the complexity of biological systems. Further, in vitro and in vivo studies are required to validate the present study.

## Supplementary Information

Below is the link to the electronic supplementary material.

Supplementary file1 (PNG 10738 KB)

Supplementary file2 (XLSX 2202 KB)

## Data Availability

No datasets were generated or analysed during the current study.
