# Supplementary figures and images for: Computational screening and molecular dynamics of natural compounds targeting the SH2 domain of STAT3: a multitarget approach using network pharmacology
Source: Mol Divers. 2025 Jan 9;29(6):5607–33. doi: 10.1007/s11030-024-11075-5 (PMC12638358; doi:10.1007/s11030-024-11075-5)

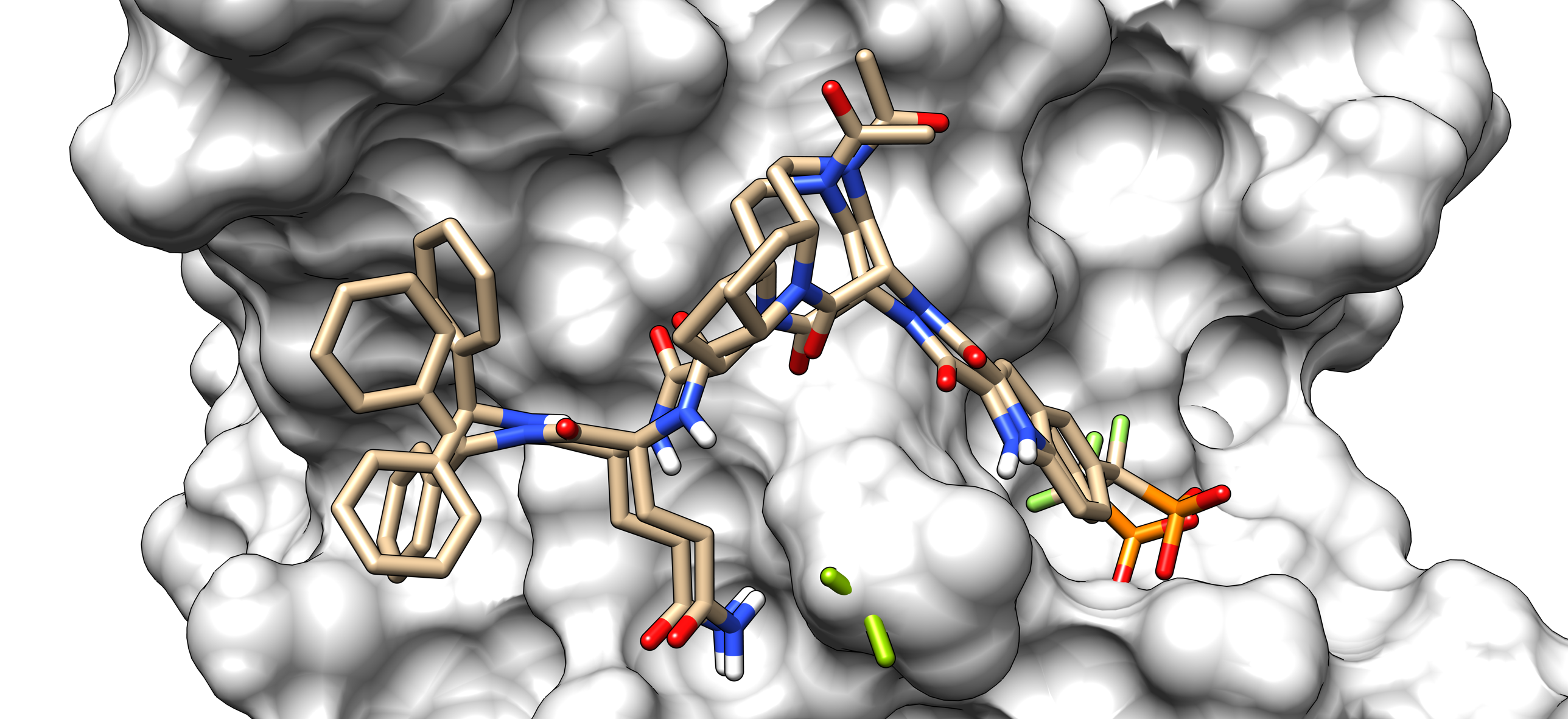

Supplement: Supplementary file 1 — Supplementary file1 (PNG 10738 KB) [file 11030_2024_11075_MOESM1_ESM.png]
